# Circulating Lymphocyte Subsets Are Associated with Diabetic Kidney Disease and Overall Survival in Patients with Type 2 Diabetes

**DOI:** 10.3390/biomedicines14051171

**Published:** 2026-05-21

**Authors:** Guanglan Li, Jiayi Chen, Chenfeng Xu, Ganyuan He, Feng Yu, Wei Liu, Yanhua Wu, Wenke Hao, Wenxue Hu

**Affiliations:** 1 Department of Nephrology, Guangdong Provincial People’s Hospital (Guangdong Academy of Medical Sciences), Guangdong Provincial Geriatrics Institute, Southern Medical University, Guangzhou 510000, China; liguanglan357@163.com (G.L.); jayee_chen@163.com (J.C.);; 2 Division of Nephrology, Department of Medicine, The Fifth Affiliated Hospital, Sun Yat-Sen University, Zhuhai 519000, China; 3 School of Medicine, South China University of Technology, Guangzhou 510000, China

**Keywords:** CD4^+^ T cells, CD8^+^ T cells, B cells, diabetic kidney disease, renal function

## Abstract

**Background:** The immune mechanism of diabetic kidney disease (DKD) has not yet been fully elucidated. This study aimed to characterize circulating lymphocyte subsets in patients with type 2 diabetes mellitus (T2DM), with a particular focus on DKD-related immune alterations and prognosis. **Methods:** Circulating T cells, B cells and NK cells were identified by flow cytometry. The primary endpoint was all-cause mortality, and overall survival was defined as the time from enrollment to death from any cause or last follow-up. Associations between lymphocyte subsets, inflammatory indices and renal function parameters were analyzed. Cox regression was used to identify factors associated with overall survival in patients with DKD and in the whole T2DM cohort. A prognostic nomogram was developed in the whole T2DM cohort to estimate 1-, 2-, 3-, and 5-year overall survival (OS) probabilities. Model performance was evaluated using the concordance index (C-index), calibration curves, receiver operating characteristic (ROC) curves, and decision curve analysis (DCA). Mendelian randomization (MR) was performed as a further exploratory analysis to assess whether immune-related traits were genetically associated with DKD susceptibility, with inverse variance weighting (IVW) as the primary analytical method. **Results:** In total, 74 T2DM patients were divided into DKD (stage 3–4 of chronic kidney disease) and non-DKD groups. Median follow-up duration was 34.6 months. DKD patients exhibited elevated levels of NK cells, the monocyte-to-lymphocyte ratio (MLR), neutrophil-to-lymphocyte ratio (NLR), and platelet-to-lymphocyte ratio (PLR). In patients with DKD, higher PLR and serum creatinine (SCr) were associated with poorer overall survival, whereas CD4^+^CD25^+^ T cell frequency was not significant after adjustment. In the whole T2DM cohort, higher frequency of circulating CD4^+^CD25^+^ T cells were associated with improved survival (HR 0.920, 95% CI 0.858–0.986, *p* = 0.019), whereas elevated PLR and SCr were linked to poorer outcomes. The exploratory nomogram incorporating CD4^+^CD25^+^ T cells, PLR, and SCr, showed acceptable internal performance in this cohort. As a separate exploratory analysis, MR suggested that genetically proxied CD4 expression on activated CD4 regulatory T cells was associated with a lower risk of DKD. **Conclusions:** DKD was associated with higher mortality and elevated MLR-, NLR-, PLR-, and NK cell levels in patients with T2DM. In patients with DKD, PLR and SCr were associated with overall survival, supporting the prognostic relevance of systemic inflammation and renal dysfunction. Individual lymphocyte subsets were not independently associated with survival in the DKD cohort after adjustment, whereas CD4^+^CD25^+^ T cell frequency provided additional prognostic information in the whole extended T2DM cohort analysis. Further validation is warranted.

## 1. Introduction

Diabetic kidney disease (DKD) is one of the main causes of chronic kidney disease (CKD) [1]. Most of patients with DKD develop into end-stage renal disease (ESRD) with poor prognosis [2], which has become a major health problem worldwide. However, the available therapies have not been completely effective in the treatment of DKD, which indicates that it is necessary to further understand the pathogenesis of DKD to improve the treatment of DKD.

The mechanisms of DKD are complex and involve the interaction of many factors. Early studies have shown that advanced glycation end products, hemodynamic changes, polyol pathway activation, protein kinase activity, and abnormal lipid metabolism are involved in the pathogenesis of DKD. Furthermore, recent studies have found new factors regarding the pathogenesis of DKD, such as immune inflammation, epithelial–mesenchymal transition, apoptosis and mitochondrial damage, epigenetics, and podocyte–endothelial communication [3]. Many immune cells play important roles in DKD, including key members of both the innate and adaptive immune systems [4]. Moon JY et al. indicated that abnormal intrarenal infiltration and activation of T cells in renal interstitium were the underlying immunopathological mechanisms of DKD [5]. Li T et al. demonstrated that B cells were associated with DKD [6]. And Zuo MH et al. found that the percentage of NK cells was lower in DKD than healthy subjects [7]. However, data on lymphocyte subsets in DKD remain scarce and incomplete. In this study, we performed a comprehensive immunophenotyping on lymphocyte subsets, and analyzed the associations of these subsets and prognosis of DKD.

The nomogram serves as a reliable and quantitative clinical tool for estimating the probability of specific outcomes, such as overall survival, by integrating multiple prognostic variables [8]. Studies have demonstrated that non-invasive diagnostic models based on clinical and laboratory parameters can effectively distinguish diabetic nephropathy from non-diabetic nephropathy in patients with type 2 diabetes mellitus (T2DM). Specifically, a multicenter study by Lin et al. developed and validated a predictive nomogram that integrated factors including diabetic retinopathy, diabetes duration, HbA1c, neutrophil-to-lymphocyte ratio (NLR), kidney volume, triglycerides, estimated glomerular filtration rate (eGFR), and urinary red blood cell count. This model showed high diagnostic accuracy (AUC > 0.92), providing a practical alternative to renal biopsy in routine clinical practice [9]. While other research has highlighted that an increased NLR was positively associated with a higher risk of adverse chronic kidney disease (CKD) progression in patients with CKD or type 2 diabetes, few studies have evaluated whether circulating lymphocyte subsets, together with inflammatory indices and renal function-related indicators, are associated with survival outcomes in patients with T2DM and diabetic kidney involvement [10].

Mendelian randomization (MR) is an analytical approach that leverages genetic variants as instrumental variables to infer causal relationships between exposures and outcomes [11]. The random allocation of genetic variants at conception, independent of the postnatal environment, minimizes confounding by factors that typically bias observational studies [12]. Furthermore, because germline genetic variants are fixed at birth and remain unchanged by subsequent disease processes, MR designs are inherently protected from reverse causation. Consequently, compared to conventional observational epidemiology, MR offers a more robust framework for causal inference by substantially reducing biases attributable to confounding and reverse causality [13]. Specifically, HLA DR to CD14^−^CD16^−^ cells, the monocytic myeloid-derived suppressor cell absolute count, the SSC-A count of CD4^+^ T cells, and terminally differentiated CD4^+^ T cells were identified as potential protective factors [14,15].

This study aimed to characterize peripheral lymphocyte subsets in patients with T2DM, with a particular focus on DKD-related immune alterations and prognosis. We first compared clinical and immune profiles between patients with and without DKD, and then examined prognostic factors for overall survival in patients with DKD. Given the limited number of death events in the DKD cohort, survival analysis was further extended to the whole T2DM cohort to evaluate the prognostic relevance of peripheral immune phenotypes across different degrees of renal involvement. MR was performed as a further exploratory analysis to assess whether immune traits were genetically associated with DKD susceptibility.

## 2. Materials and Methods

### 2.1. Study Population of Observational Analysis

A total of 74 adult patients with diabetes were recruited in Guangdong Provincial People’s Hospital from March 2010 to December 2021, and 39 patients were diagnosed with DKD (stage 3–4 of Chronic kidney disease). Based on the 2012 Kidney Disease Improving Global Outcomes (KDIGO) guidelines, DKD was diagnosed when either of the following criteria was met: (1) Albumin/creatinine ratio (ACR) > 300 mg/g, or albumin > 300 mg/24 h; (2) microalbumin (ACR was between 30 and 300 mg/g or albumin was between 30 and 300 mg/24 h) and estimate glomerular filtration rate (eGFR) > 30 mL/min/1.73 m^2^. eGFR values were calculated by using the Chronic Kidney Disease Epidemiology Collaboration equation (CKD-EPI) [16]. Exclusion criteria: (1) Patients receiving kidney transplantation; (2) patients with isolated kidney; (3) patients with acute infection; (4) patients with malignant tumor; (5) patients with autoimmune diseases. Chronic kidney disease (CKD) was diagnosed and staged according to the KDIGO guidelines [17]: stage 2 was defined as an eGFR from 60 to 89 mL/min/1.73 m^2^, stage 3 was defined as an eGFR from 30 to 59 mL/min/1.73 m^2^, and stage 4 was defined as an eGFR from 15 to 29 mL/min/1.73 m^2^.

For all patients, we recorded clinical data, including age and sex, hypertension status, duration of diabetes, hemoglobin (Hb), white blood cells, neutrophil, lymphocytes, monocytes, platelets, immunoglobulin A (IgA), immunoglobulin G (IgG), immunoglobulin M (IgM), glycosylated hemoglobin (HbA1c), serum albumin (Alb), urinary β2 microglobulin, uric acid, serum creatinine (SCr), blood urea nitrogen (BUN), Cystatin C, estimated glomerular filtration rate (eGFR) and albumin/creatinine ratio (ACr). The neutrophil-to-lymphocyte ratio (NLR), monocyte-to-lymphocyte ratio (MLR), and platelet-to-lymphocyte ratio (PLR) were calculated. The study involving human participants was approved by the Ethical Committee of Guangdong Provincial People’s Hospital (Approval number: KY-Q-2021-276; Date of approval: 14 March 2022). Informed consent has been obtained from the subjects.

### 2.2. Clinical Outcome of Observational Analysis

The primary endpoint of this study was all-cause mortality. Overall survival (OS) was defined as the time from enrollment to death from any cause or last follow-up. All patients were followed until the date of their last visit, and the median follow-up duration was 34.6 months.

### 2.3. Flow Cytometry Analysis of Observational Analysis

**Figure 1:**
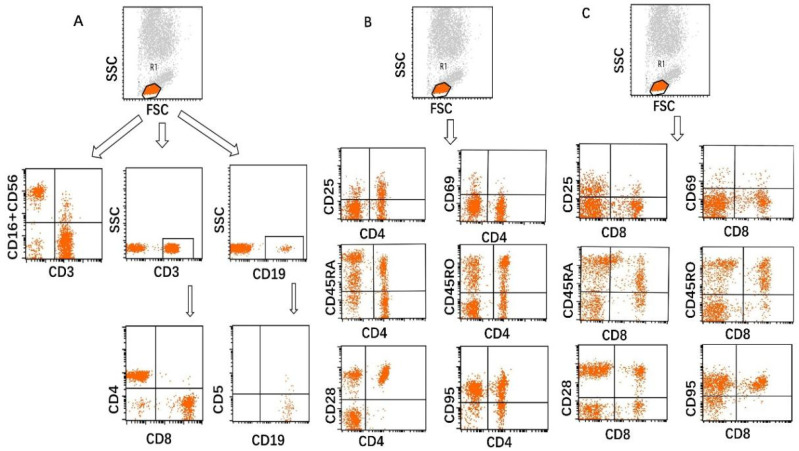
Gating strategies of lymphocyte phenotype analysis. (**A**): Lymphocytes were gated as R1 based on their forward and side scatter characteristics (FSC/SSC). From this R1 gate, Lymphocyte subsets were identified by B cells (CD19^+^CD5^+^ and CD19^+^CD5^−^), T cells (CD3^+^CD4^+^ and CD3^+^CD8^+^), and NK cells (CD3-CD16^+^CD56^+^). (**B**): CD4^+^ T cells were further identified by CD25^+^, CD69^+^, CD45RA^+^, CD45RO^+^, CD28^+^, and CD95^+^. (**C**): CD8^+^ T cells were further identified by CD25^+^, CD69^+^, CD45RA^+^, CD45RO^+^, CD28^+^, and CD95^+^.

Studies were performed on cells remaining after red blood cell lysis of diluted whole blood that had undergone antibody immunostaining. Lymphocytes were identified by side scatter (SSC) and forward scatter (FSC) properties. A total of 100 µL of whole blood was incubated with 10 µL of anti-human CD3-PerCP, CD4-FITC, CD8-PE, CD16^+^CD56PE, CD19-APC, and CD5-PE antibodies for 20min at 4 °C in the dark. B lymphocytes were further identified by CD19^+^, CD5^+^ immunostaining, T lymphocytes were further identified by CD3^+^, CD4^+^, and CD8^+^ immunostaining, NK cells were further identified by CD3^−^, CD16^+^, and CD56^+^ immunostaining. For lymphocyte phenotype analysis, monoclonal antibodies against CD25, CD69, CD45RA, CD45RO, CD28, and CD95 were used according to the manufacturer’s instructions. Additional immunostaining was performed on CD4^+^ and CD8^+^ T lymphocytes to identify the following: (1) CD25: the interleukin-2 receptor alpha chain expressed on activated T cells and regulatory T cells [18]; (2) CD69: a marker of cell activation [19]; (3) CD45RA: a marker of Naïve T cells [20]; (4) CD45RO: a marker of memory T cells [20]; (5) CD28: a key positive costimulatory molecule [21]; (6) CD95: a member of the tumor necrosis factor receptor [22]. Samples were analyzed with FACSCanto flow cytometer (BD Biosciences, San Jose, CA, USA), using FACSCanto II (BD Biosciences, San Jose, CA, USA). CD4^+^CD25^+^ T cells were defined as the proportion of CD25-positive cells within the CD3^+^CD4^+^ T cell gate. The threshold for CD25 positivity was determined according to the corresponding negative population/control and was applied consistently across samples. CD25 expression was analyzed as a positive/negative marker, and CD25 high and CD25 intermediate subsets were not separately quantified in this study. Representative gating plots are shown in Figure 1.

### 2.4. Statistical Analysis of Observational Analysis

Statistical analyses were conducted in R (version 4.2.2), utilizing packages including “ggDCA”, “survival”, “rms”, “pROC”, and “nomogramFormula”. Normally distributed data were presented as means ± standard deviation and non-normally distributed data were presented as medians and interquartile ranges (IQRs). Continuous variables were compared by using the Student *t* test or Mann–Whitney U test. Categorical variables were compared by using the χ^2^ test. Pearson’s or Spearman’s test were used for the correlation between lymphocyte subsets and clinical data.

For survival analyses, univariate Cox proportional hazard regression was first performed to screen candidate variables. In the DKD-focused survival analysis, variables with *p* < 0.10 in univariate Cox regression were considered candidate variables for subsequent selection, in order to avoid excluding prematurely potentially relevant predictors in this small DKD cohort. This threshold was used only for candidate screening, while statistical significance in the final multivariable Cox model was defined as *p* < 0.05. Candidate variables were further assessed using variance inflation factor (VIF) analysis to evaluate multicollinearity and least absolute shrinkage and selection operator (LASSO)-Cox regression to select prognostic variables. The selected variables were then entered into multivariable Cox proportional hazard regression. Hazard ratios (HRs) and 95% confidence intervals (CIs) were calculated to estimate the association of each factor with overall survival.

For the extended analysis in the whole T2DM cohort, a prognostic nomogram was then developed based on the results of multivariate Cox analysis, refined through a backward stepwise selection process guided by the Akaike information criterion (AIC). The X-tile software version 3.6.1 (Yale University, New Haven, CT, USA) was used to determine the optimal cutoff point of the nomogram-derived risk score for stratifying patients into low- and high-risk subgroups to evaluate model discrimination. Survival outcomes between risk groups were compared using Kaplan–Meier curves and the log-rank test. Model validation encompassed assessments of both discrimination and calibration. Discrimination was evaluated using the concordance index (C-index) and time-dependent receiver operating characteristic (ROC) curves at 1, 2, 3, and 5 years. The C-index ranges from 0.5 (no discrimination) to 1.0 (perfect discrimination). Calibration was performed by comparing the nomogram-predicted survival probabilities with observed Kaplan–Meier survival estimates across risk deciles. Furthermore, decision curve analysis (DCA) was employed to evaluate the potential clinical utility of the nomogram by quantifying its net benefit across a range of threshold probabilities. All statistical tests were two-sided, and a *p*-value < 0.05 was considered statistically significant unless otherwise specified.

### 2.5. Data Sources in Mendelian Randomization (MR) Analysis

Immunology-related GWAS data sources in our research were derived from open GWAS databases. The studies involved have all been approved by the local ethics committee. This study did not collect new data and does not require new ethical approval. A cohort study involving 3757 Sardinian individuals reported data on 22 million variants for 731 immune cell phenotypes (from ebi-a-GCST90001391 to ebi-a-GCST0002121), comprising immunophenotyping measurements of surface antigen expression on CD4^+^ T cells. This analysis included: (1) baseline expression on total CD4^+^ T cells (ebi-a-GCST90002022, ebi-a-GCST90001960); (2) expression distinguishing regulatory T cells (ebi-a-GCST90001936), Naïve conventional (ebi-a-GCST90001934), and effector conventional T cells (ebi-a-GCST90001933); (3) expression across Treg functional states: resting (ebi-a-GCST90001937), secreting (ebi-a-GCST90001941), and activated (ebi-a-GCST90001939, ebi-a-GCST90002066); and (4) expression of activation markers on HLA DR+ CD4^+^ T cells (ebi-a-GCST90001959, ebi-a-GCST90002114) as a control for nonspecific T cell activation.

The DKD data were obtained from the GWAS database (https://www.ebi.ac.uk/gwas/studies/GCST90018832, accessed on 15 May 2026), comprising a study population of 452,280 individuals of European descent. The case group consisted of 1032 individuals, and the control group included 451,248 individuals, involving 24,190,738 SNPs.

### 2.6. Instrumental Variables (IVs) in MR Analysis

In this Mendelian Randomization (MR) study, single nucleotide polymorphisms (SNPs) served as instrumental variables (IVs). Valid IVs were required to meet three core assumptions: (1) a robust association with the exposure, (2) independence from known or unknown confounders, and (3) an effect on the outcome mediated exclusively through the exposure. For immune-related exposures, IVs were selected at a genome-wide significance level (*p* < 5 × 10^−8^). To ensure independence, SNPs were clumped using a 10,000 kb window and a linkage disequilibrium (LD) threshold of r^2^ < 0.001. Palindromic SNPs with ambiguous strand orientation were resolved through allele frequency harmonization, ensuring reliable data integration while maximizing variant retention.

### 2.7. Statistical Analysis of MR Analysis

**Figure 2:**
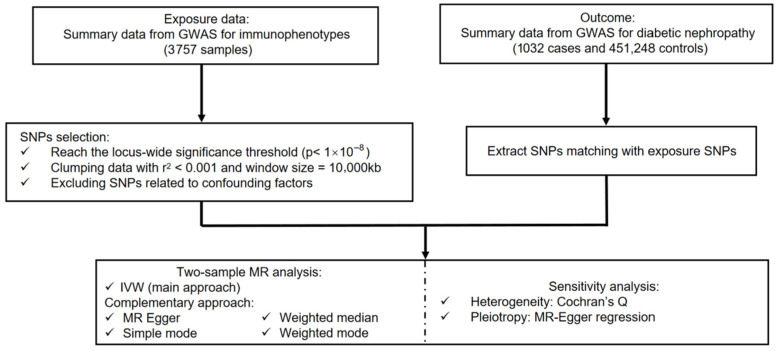
Flowchart illustrating the present MR study. GWAS, genome-wide association study; SNPs, single nucleotide polymorphisms; IVW, inverse variance weighted.

Statistical analyses were conducted in R (version 4.2.2), utilizing packages including “TwoSampleMR”, “MRInstruments” and “MRPRESSO” packages. In this study, we employed multiple analytical methods for MR analyses. Initially, immune cell phenotypes were examined as exposures to identify potential causal effects on the risk of DKD. A reverse-direction MR analysis to evaluate potential causal effects of DKD on immune cell phenotypes was considered. However, the number of genome-wide significant SNPs available for DKD as an exposure was insufficient to construct a robust set of instrumental variables, precluding a statistically reliable analysis in this direction. The flowchart of the two-sample MR analysis for immune cell phenotypes and the risk of DKD is depicted in Figure 2.

To assess the potential bidirectional causal relationship between immune cell phenotypes and DKD, we employed multiple MR methods. The primary causal estimate was derived using the inverse-variance weighted (IVW) method, which yields an efficient pooled estimate under the assumption of balanced pleiotropy. Robustness was assessed through four supplementary methods: (1) MR-Egger regression, which provides an estimate corrected for directional pleiotropy; (2) the weighted median method, which remains consistent if at least 50% of the instrumental variables are valid; (3) the simple mode method, which identifies the most common point estimate among SNPs; and (4) the weighted mode method, a weighted version of the simple mode that is less sensitive to outliers. Horizontal pleiotropy was evaluated via the MR-Egger intercept test, and heterogeneity across genetic instruments was quantified using Cochran’s Q statistic, with statistical significance defined as *p* < 0.05.

## 3. Results

### 3.1. The Characteristics of Patients

In our study, a total of 74 patients with T2DM were enrolled at Guangdong Provincial People’s Hospital between March 2010 and December 2021, including 39 (52.7%) DKD patients (31 males and 8 females). The median follow-up time was 34.6 months (range, 1.1–140.0 months). The 1-, 2-, 3-, and 5-year all-cause mortality rates were significantly higher in patients with DKD than in those without DKD. During the entire follow-up period, 24 all-cause death events occurred among the 74 patients with type 2 diabetes, corresponding to an overall event rate of 32.4%. Specifically, 15 deaths occurred in the DKD group (15/39, 38.5%) and 9 deaths occurred in the non-DKD group (9/35, 25.7%). DKD patients were significantly older than controls. The values of hemoglobin, lymphocytes, Alb and eGFR were smaller in DKD patients than those in non-DKD patients. In contrast, the values of IgM, urinary β2 microglobulin, SCr, BUN, Cystatin C and ACr were much higher in DKD patients than those in non-DKD patients. No statistically significant differences were found in HbA1C, IgA or IgG. The clinical characteristics are summarized in Table 1.

### 3.2. The Correlation Between Lymphocyte Subsets and Clinical Data

**Figure 3:**
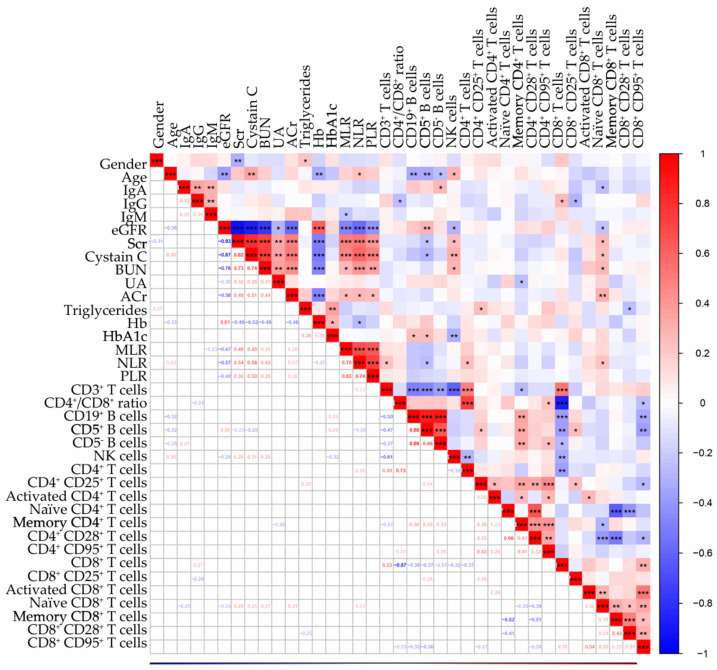
Correlation analysis between clinical characteristics. * *p* < 0.05, ** *p* < 0.01, *** *p* < 0.001.

Lymphocyte subsets were identified by flow cytometry analysis. DKD patients presented higher levels of MLR, NLR, PLR and NK cells (*p* < 0.05, Table 2). No significant differences were observed in the levels of CD3^+^ T cells (including CD3^+^CD4^+^ T cells and CD3^+^CD8^+^ T cells) or CD19^+^ B cells (including CD19^+^CD5^+^ B cells and CD19^+^CD5- B cells) between the two groups. Correlation analysis revealed that B cells were negatively associated with age, whereas NLR and NK cells were positively associated with age (Figure 3). CD19^+^ B cells and CD5^+^ B cells were positively correlated with HbA1c, contrasting with an inverse association for NK cells. No lymphocyte subsets associated with hemoglobin or gender.

And we further identified CD4^+^ and CD8^+^ T cell subsets. For CD4^+^ T cell subsets, DKD patients presented higher levels of CD4^+^CD25^+^ T cells, CD4^+^ CD28^+^ T cells, CD4^+^CD95^+^ T cells and lower levels of activated CD4^+^ T cells (CD4^+^CD69^+^), Naïve CD4^+^ T cells (CD4^+^CD45RA^+^), and memory CD4^+^ T cells (CD4^+^CD45RO^+^) without significant difference (*p* > 0.05). Memory CD4^+^ T cells inversely associated with uric acid, while CD4^+^CD25^+^ T cells positively correlated with triglycerides.

CD8^+^ T cell subsets were similar to CD4^+^ T cell subsets. DKD patients presented higher levels of CD8^+^CD25^+^ T cells, activated CD8^+^ T cells (CD8^+^CD69^+^), Naïve CD8^+^ T cells (CD8^+^CD45RA^+^), memory CD8^+^ T cells (CD8^+^CD45RO^+^), CD8^+^CD95^+^ T cells, and lower levels of CD8^+^CD28^+^ T cells without significant difference (*p* > 0.05).

Focusing on renal function parameters, correlation analysis revealed that MLR, NLR, PLR, NK cells, and Naïve CD8^+^ T cells were negatively associated with eGFR and positively associated with SCr, Cystatin C, BUN and ACr. Conversely, CD5^+^ B cells exhibited renal-protective associations, positively correlating with eGFR and negatively with SCr and Cystatin C. For immunoglobulin, we found that IgA was positively related to CD5^−^ B cells and negatively associated with Naïve CD8^+^ T cells. IgG was positively related to CD8^+^ T cells and negatively associated with CD4/CD8 ratio and CD8^+^CD25^+^ T cells. IgM inversely correlated with MLR.

### 3.3. Prognostic Factors for Overall Survival in Patients with DKD

We explored the prognostic relevance of clinical indicators and lymphocyte subsets within the DKD subgroup. The demographic features (age, gender, hypertension and duration of diabetes), clinical features demonstrating significant intergroup differences, and lymphocyte phenotypes were subjected to univariate Cox regression analysis. The analysis identified six candidate variables associated with survival outcomes at *p* < 0.1 (Table 3): NLR, PLR, SCr, BUN, eGFR, and CD4^+^CD25^+^ T cells. Prior to multivariable analysis, variance inflation factor (VIF) assessment was performed to detect potential multicollinearity among these 6 variables. The analysis revealed significant collinearity for SCr (VIF = 7.384) and eGFR (VIF = 7.337), exceeding the conservative threshold for multivariable Cox regression (Table 4).

**Figure 4:**
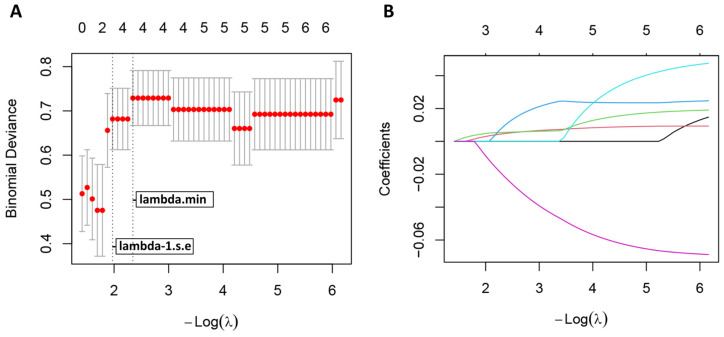
LASSO-based selection of candidate prognostic variables in patients with DKD. (**A**) A total of 6 risk factors selected using LASSO regression analysis. The optimal tuning parameter (λ) was determined via ten-fold cross-validation based on the minimum criteria. The vertical dashed lines indicate the optimal λ values selected by the minimum criteria and the one-standard-error (1-SE) rule, which provides the most parsimonious model within one standard error of the minimum. (**B**) LASSO coefficient profiles of the 6 features. A coefficient profile plot was produced against the log (λ) sequence. At the 1-SE rule including PLR, SCr and CD4^+^CD25^+^ T cells. Each colored line represents one of the 6 candidate variables, with distinct colors used to differentiate them.

To address this limitation and identify the most optimal predictive features, we employed the LASSO-Cox regression model. Among the 6 candidate variables, LASSO analysis selected 3 features that demonstrated the strongest prognostic value: PLR, SCr and CD4^+^CD25^+^ T cells (Figure 4). Subsequent VIF confirmation established that all three selected variables exhibited values well below the conservative threshold of 5, ensuring variable independence for subsequent analyses. These three LASSO-selected features were subsequently incorporated into a multivariable Cox regression model. The final model results are presented in Table 3. In patients with DKD, higher PLR (HR 1.009, 95% CI 1.002–1.016, *p* = 0.013) and higher serum creatinine (HR 1.009, 95% CI 1.000–1.019, *p* = 0.047) were significantly associated with poorer overall survival. Conversely, CD4^+^CD25^+^ T cell frequency showed an HR below 1, but the association did not reach statistical significance in multivariable Cox regression (HR 0.931, 95% CI 0.848–1.023, *p* = 0.136).

### 3.4. Extended Prognostic Modeling in the Whole T2DM Cohort

The initial DKD-focused survival analysis was limited by the relatively small number of patients and death events, and most lymphocyte subsets did not show robust prognostic associations. Since all participants had T2DM and all-cause death events occurred in both DKD and non-DKD patients, survival analysis was further extended to the whole T2DM cohort to evaluate the prognostic relevance of peripheral immune phenotypes across different degrees of renal involvement.

In the whole T2DM cohort, univariate Cox regression followed by LASSO-based feature selection and multivariable Cox regression identified CD4^+^CD25^+^ T cells, PLR, and SCr as factors associated with overall survival (Table 5, Supplementary Tables S1–S3 and Supplementary Figure S1). Among all peripheral blood lymphocyte and lymphocyte subsets analyzed, only higher levels of CD4^+^CD25^+^ T cells (HR 0.920, 95% CI 0.858–0.986, *p* = 0.019) were significantly associated with better prognosis in T2DM. Conversely, patients with lower PLR (*p* = 0.017) and lower serum creatinine levels (*p* < 0.001) demonstrated significantly longer survival in our cohort.

**Figure 5:**
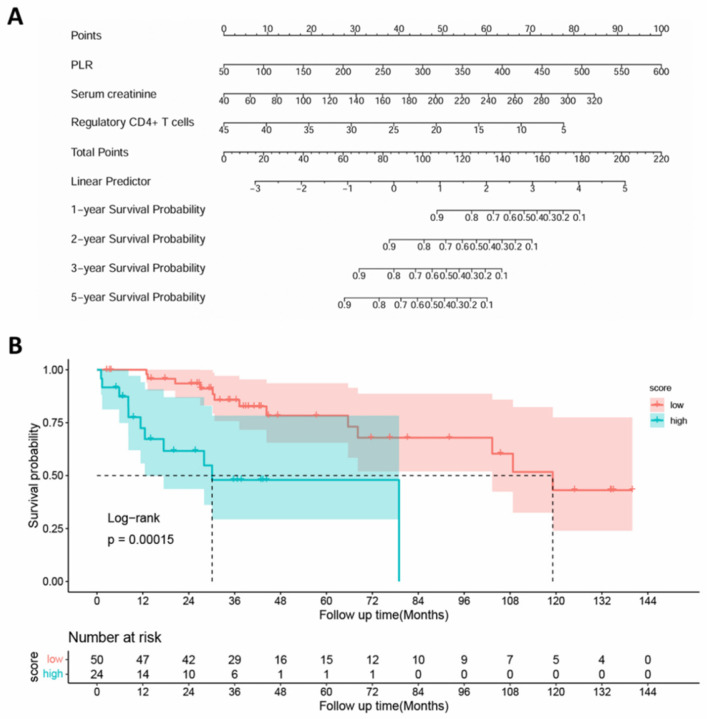
Extended nomogram and risk stratification for overall survival in the whole T2DM cohort. (**A**) Prognostic nomogram for patients with T2DM. Each variable was assigned a specific score on the point scale. By summing the total scores of all variables and locating the aggregate value on the total points scale, the corresponding 1-, 2-, 3-, and 5-year survival probabilities could be estimated by drawing a vertical line downward to the survival probability axes. (**B**) Overall survival in the subgroup according to cutoff value (101.53) of the total score.

Based on these three predictors, an extended nomogram was constructed to estimate 1-, 2-, 3-, and 5-year overall survival probabilities in patients with T2DM (Figure 5A). In the nomogram, PLR contributed most substantially to risk prediction, followed by SCr and CD4^+^CD25^+^ T cell frequency. The model showed good internal discrimination, with a bootstrap-corrected AUC of 0.894 (95% CI 0.815–0.973), and the calibration, time-dependent ROC, bootstrap validation, and decision curve analyses are shown in Supplementary Figure S2. Using X-tile software 3.6.1, a total score of 101.53 was identified as the optimal cutoff value, and patients were stratified into low- and high-risk groups (Figure 5B). The high-risk group showed shorter overall survival and a higher prevalence of DKD. In addition, patients in the high-risk group had poorer renal function, higher inflammatory indices, and lower hemoglobin and albumin levels than those in the low-risk group (Table 6). These results further indicate that the whole-cohort model captured a mortality-risk gradient closely related to DKD involvement.

### 3.5. Further Exploratory Analysis of Immune-Related Traits and DKD Susceptibility

Because all participants in the observational cohort had T2DM, this study focused on immune alterations related to diabetic kidney involvement rather than immune susceptibility to T2DM itself. The observational analyses evaluated associations between circulating lymphocyte subsets and overall survival, whereas the MR analysis addressed a different question: whether genetically proxied immune traits were associated with DKD susceptibility. Therefore, MR was performed as a separate exploratory analysis using DKD as the outcome.

Our clinical cohort analysis showed that a higher level of peripheral CD4^+^CD25^+^ T cells was associated with improved overall survival in patients with T2DM. However, this immunophenotype is biologically heterogeneous, encompassing both immunosuppressive regulatory T cells (Tregs) and activated effector conventional T cells, which often exert opposing functions [23]. In this context, Treg-related terminology in the MR analysis refers to regulatory T cell-related immune traits and should not be directly equated with the CD4^+^CD25^+^ T cell population measured by flow cytometry in our clinical cohort. We employed a two-sample MR framework using genetic instruments for CD4 or CD25 surface expression on distinct immune cell subsets to further explore whether regulatory T cell-related immune traits were genetically associated with DKD susceptibility (Table 7). This approach allows us to differentiate the causal effects stemming from: (1) Tregs (using QTLs for CD25 on CD4 regulatory T cells), from (2) activation of conventional T cells (using QTLs for CD25 on non-regulatory CD4 T cells). Furthermore, we analyzed substates of Tregs (resting, activated, secreting) to investigate functional relevance, and controlled for general T cell activation status using HLA-DR expression.

**Figure 6:**
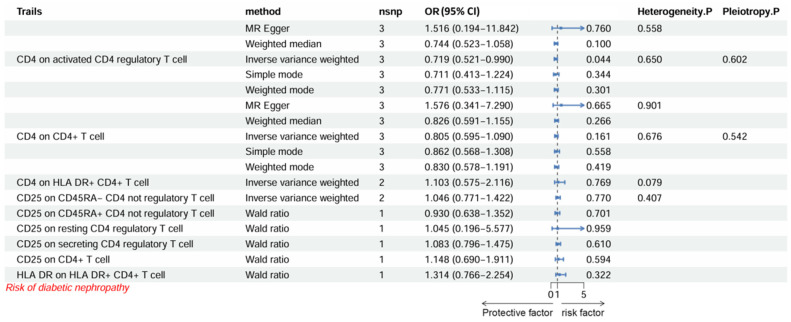
Causal effects of immune cell phenotypes on DKD risk. OR, odds ratio; CI, confidence interval; nsnp, number of single nucleotide polymorphisms (instrumental variables).

We first analyzed 11 immune cell phenotypes as exposure variables, with DKD as the outcome. The results are shown in Figure 6. The MR analysis suggested that genetically proxied CD4 on activated CD4 regulatory T cells was negatively associated with DKD risk (OR = 0.719, 95% CI = 0.521–0.990, *p* = 0.044, IVW method). No evidence of horizontal pleiotropy was observed, and Cochran’s Q test indicated no significant heterogeneity across the genetic instruments. Reverse MR analysis from DKD to immune cell phenotypes was not performed, because the limited number of SNPs would not support reliable causal estimation or sensitivity analyses. The number of SNPs retained for each immune phenotype after LD clumping and harmonization, together with the corresponding F statistics, is provided in Supplementary Table S4.

## 4. Discussion

An extraordinarily high rate of diabetes predicts a commensurate increase in the rate of diabetic complications, particularly DKD. And DKD is one of the major causes of ESRD throughout the world in both developed and developing countries, which is a growing challenge for health care systems [24]. Increasing evidences suggest that immunity and inflammation play a paramount role in the pathogenesis of DKD. The cells of the adaptive immune system consist of helper (CD4^+^) T cells, cytotoxic (CD8^+^) T cells, and B cells, which can be activated during the immune response and differentiate into different cell phenotypes according to the immune environment [25]. Recent reports have shown that T and B cells infiltrate the kidney in animal DKD models [26]. Up to now, there have been few studies on the role of NK cells in DKD. One study found that the percentage of NK cells was lower in DKD than healthy subjects, but there was no change in the percentage of NK cells after treatment with glutathione in DKD patients [7]. However, there are few studies on the relationship between lymphocyte subsets and prognosis of DKD.

People with diabetes who develop kidney disease are at increased risk of mortality [27], which was consistent with our results. In our study, the mortality was higher in DKD patients than that in non-DKD patients within five-year data. And our results also showed that DKD patients exhibited higher levels of MLR-, NLR-, PLR and NK cells, but there was no statistical difference in T cells or B cells between the two groups. In addition, statistically elevated NK cell levels were observed both in patients with DKD and in those identified as high-risk by the nomogram, positively associating with poor renal function, which contrasted with the previously reported decrease [7]. The differences observed may be partially explained by the presence of a considerable proportion of non functional NK cells mixed in peripheral blood. Therefore, the role of NK cells in DKD patients needs to be further studied.

On antigen encounter, Naïve CD4^+^ and CD8^+^ T cells differentiate into large numbers of activated T cells, and CD28 is an important costimulatory molecule of T cell activation [21]. Following elimination of the antigen, some cells remain in the long-term, the so-called memory T cells, which are able to re-expand and respond more forcefully on a second encounter with the cognate antigen [28]. CD95 (also known as Fas) is the prototype of death receptors [29], which is required for programmed cell death after T cell activation [30,31]. Our data revealed no statistically significant differences (*p* > 0.05) between DKD patients and controls in the proportions of CD4^+^ or CD8^+^ T cell subsets. These findings suggest that broad changes in circulating T cell differentiation phenotypes may not be the dominant feature distinguishing DKD status.

In patients with DKD, PLR and SCr were the main factors associated with overall survival after adjustment. CD4^+^CD25^+^ T cell frequency was not independently significant in the DKD cohort, but provided additional prognostic information in the extended whole T2DM cohort analysis. Higher circulating CD4^+^CD25^+^ T cell frequency was associated with better overall survival, while higher PLR and SCr were associated with poorer outcomes. These findings suggest that CD4^+^CD25^+^ T cell frequency may provide additional prognostic information in the broader T2DM population. The interpretation of CD4^+^CD25^+^ T cells requires caution. CD25 is expressed not only on regulatory T cells (Tregs) but also on activated conventional T cells. Because our flow cytometry panel did not include Foxp3 or CD127, the measured CD4^+^CD25^+^ T cell population cannot be equated with Tregs. Thus, the observed association should be interpreted as reflecting peripheral immune status rather than kidney-specific Treg function. As a separate exploratory analysis, MR suggested that genetically proxied CD4 expression on activated CD4 regulatory T cells was associated with a lower risk of DKD. This MR result addresses DKD susceptibility, whereas the clinical cohort analysis addresses overall survival; therefore, the two analyses are complementary but not equivalent. Treg cells are crucial for maintaining immune homeostasis. Forkhead box P3 (FOXP3) is a key transcription factor in Treg cell homeostasis and can be degraded by pro-inflammatory cytokines such as TNF-α in chronic inflammation [32]. This finding is further supported by prior research indicating that CD4(+)FoxP3(+) Tregs ameliorate insulin resistance and renal injury via suppressing inflammatory responses, highlighting their central role in glucose metabolism and kidney protection [33]. Our results, supporting earlier evidence [34], find no correlation between Treg levels and diabetes duration, systemic inflammation, or disease progression in T2DM. Moreover, the observed positive correlation between CD4^+^CD25^+^ T cells and triglycerides may be attributed to the dual role of regulatory T cells in modulating both immune suppression and metabolic homeostasis.

For all CD8^+^ T cell subsets, no significant differences were observed between DKD patients and controls. However, we also observed increased Naïve CD8^+^ T cells were positively associated with worse renal function. Consistent with this, stratification based on nomogram risk scores showed that high-risk patients had significantly elevated urinary β2-microglobulin, SCr, BUN, Cystatin C, ACr, and Naïve CD8^+^ T cells. Moon JY et al. demonstrated in diabetic nephropathy mouse models that T cells infiltrate the kidney and produce IFN-γ and TNF-α, and that infiltrated T cells are associated with proteinuria [5]. The increase in Naïve CD8^+^ T cells observed in our cohort may reflect their ongoing activation and recruitment into renal tissue during DKD progression, thereby contributing to inflammatory injury and functional decline. The nomogram may provide preliminary risk stratification information; however, its clinical applicability requires external validation in larger prospective cohorts.

By far, there are limited data on the role of B cells in DKD. Xiao X et al. showed infiltration of B cells into the glomeruli of type 1 non-obese diabetic mouse model [26]. And Moon JY et al. showed infiltration of CD20^+^ B cells into the interstitium of patients with type 2 diabetes, and the numbers of these cells correlated with the amount of proteinuria. In our cohort, although the difference did not reach statistical significance, circulating CD19^+^CD5^+^ B cells tended to be lower in DKD patients and showed a significant negative correlation with poor renal function, which is consistent with our previous results in CKD patients [35]. Compared to the low-risk group, patients classified as high-risk by the nomogram in our study showed significant reductions in Hb, Alb, eGFR, lymphocytes, and the frequency of CD5^+^ B cells. This decrease may be due to recruitment of B cells to the kidney, or it may be due to the absolute reduction of B cells in DKD owing to the inhibition of bone marrow function by toxin [36]. The potential pathogenesis of B cells in diabetic nephropathy remains unclear. A study demonstrated that B cells regulate immune response and inflammation by producing immunoglobin and inducing T cell activation and proliferation through antigen presentation [37]. But we noted no association between IgA, IgG, IgM and CD19^+^CD5^+^ B cells. More studies are needed to analyze the mechanism of B cells in DKD.

Our study has several limitations. First, this was a single-center retrospective study with a relatively small sample size and a limited number of death events, which may affect the stability of the Cox models and the exploratory nomogram. Second, DKD was diagnosed mainly according to clinical criteria, and kidney biopsy data were not available for most patients; therefore, renal histopathological changes and local immune infiltration could not be evaluated. Third, the primary endpoint was all-cause mortality rather than a kidney-specific endpoint. Kidney-specific outcomes, such as longitudinal eGFR decline, doubling of serum creatinine, progression to ESKD, or initiation of dialysis, were not consistently available in this retrospective cohort. Detailed treatment information and inflammatory biomarkers, such as CRP and cytokines, were not consistently available, which limited the assessment of residual confounding and underlying mechanisms. Finally, the MR analysis was exploratory and limited by the availability of immune phenotypes and the use of European-ancestry GWAS data. Larger prospective studies with external validation are needed to confirm these findings.

## 5. Conclusions

In conclusion, patients with DKD showed higher mortality and elevated inflammatory indices and NK cell levels compared with those without DKD. In the DKD cohort, PLR and SCr were associated with overall survival, highlighting the prognostic relevance of systemic inflammation and renal dysfunction. Circulating CD4^+^CD25^+^ T cells were not independently significant in patients with DKD, but provided additional prognostic information in the whole extended T2DM cohort analysis. These findings suggest that systemic inflammation and renal dysfunction are closely related to survival risk in patients with DKD, while peripheral immune status may provide additional prognostic information in the broader T2DM cohort. Further studies are needed to validate these findings and explore their specific pathogenesis and therapeutic implications.

## Figures and Tables

**Table 1:** Patients’ characteristics.

Variables	DKD Group	Non-DKD Group	*p* Value
Cases	39	35	
Age (years)	86.0 (82.5, 88.0)	80.0 (74.0, 83.5)	0.001 *
Men, *n* (%)	31 (79.5)	29 (82.9)	0.942
Hypertension, *n* (%)	35 (89.7)	33 (94.30)	0.413
Duration of diabetes (years)	11.0 (10.0, 20.0)	10.0 (3.50, 20.0)	0.115
Hb (g/L)	112 ± 19.8	133 ± 14.6	<0.001 *
White blood cells (10^9^/L)	5.89 (5.54, 7.06)	6.31 (5.49, 7.32)	0.681
Neutrophil (10^9^/L)	3.84 (3.30, 4.72)	3.81 (3.17, 4.90)	0.673
Lymphocytes (10^9^/L)	1.35 (1.17, 1.74)	1.72 (1.37, 2.10)	0.010 *
Monocytes (10^9^/L)	0.50 (0.44, 0.63)	0.49 (0.41, 0.61)	0.439
Platelets (10^9^/L)	194 (154, 242)	179 (153, 218)	0.404
IgA (g/L)	2.39 (1.88, 3.43)	2.87 (1.46, 3.59)	0.845
IgG (g/L)	11.8 (10.8, 13.8)	12.2 (10.2, 14.2)	0.799
IgM (g/L)	0.63 (0.48, 0.98)	0.52 (0.32, 0.81)	0.037 *
HbA1c (%)	7.30 (6.20, 7.90)	6.60 (6.15, 7.65)	0.404
Alb (g/L)	34.0 ± 3.17	36.3 ± 3.05	0.002 *
Urinary β2 microglobulin (mg/L)	3.27 (0.68, 16.3)	0.33 (0.12, 1.24)	<0.001 *
Uric acid (μmol/L)	388 ± 105	358 ± 83.8	0.170
SCr (μmol/L)	137 (90.2, 196)	74.3 (69.3, 84.0)	<0.001 *
BUN (mmol/L)	10.5 (6.50, 14.4)	5.08 (4.35, 6.22)	<0.001 *
Cystatin C (mg/L)	2.18 (1.43, 2.81)	1.09 (0.92, 1.33)	<0.001 *
eGFR _CKD-EPI_ (ml/min/1.73 m^2^)	40.3 (25.6, 61.6)	83.7 (72.9, 88.5)	<0.001 *
ACr (mg/g Cr)	273 (63.8, 1225)	12.3 (4.96, 31.6)	<0.001 *
All-cause mortality, *n* (%)			
1 years	6 (15.4%)	0 (0.00%)	0.026 *
2 years	10 (25.6%)	1 (2.86%)	0.015 *
3 years	14 (35.9%)	3 (8.57%)	0.012 *
5 years	14 (35.9%)	4 (11.4%)	0.029 *

DKD: diabetic kidney disease; Hb, hemoglobin; IgA, immunoglobulin A; IgG, immunoglobulin G; IgM, immunoglobulin M; HbA1c, glycosylated hemoglobin; Alb, serum albumin; SCr, serum creatinine; BUN, blood urea nitrogen; ACr, albumin-to-creatinine ratio; eGFR, estimated glomerular filtration rate; * *p* < 0.05.

**Table 2:** Comparison of lymphocyte subsets and inflammatory ratios between DKD patients and controls.

Variables	All (*n* = 74)	DKD Group (*n* = 39)	Non-DKD Group (*n* = 35)	*p* Value
MLR	0.34 (0.26, 0.47)	0.37 (0.31, 0.50)	0.29 (0.23, 0.39)	0.011 *
NLR	2.50 (1.86, 3.77)	2.98 (2.08, 4.31)	2.24 (1.67, 2.72)	0.007 *
PLR	119 (91.2, 160)	148 (96.9, 208)	108 (87.1, 129)	0.003 *
CD3^+^ T cells	70.5 (65.9, 76.9)	69.6 (65.6, 75.6)	73.9 (66.5, 77.1)	0.381
CD19^+^ B cells	7.00 (3.86, 11.5)	7.38 (3.45, 11.4)	6.85 (4.64, 12.8)	0.338
NK cells	16.4 (13.8, 23.2)	18.3 (14.0, 25.4)	15.1 (13.5, 18.0)	0.038 *
CD5^+^ B cells	1.50 (0.69, 3.89)	1.45 (0.69, 2.88)	1.89 (0.74, 4.72)	0.188
CD5^−^ B cells	4.78 (3.00, 8.53)	5.23 (2.68, 8.48)	4.62 (3.33, 8.22)	0.782
CD4^+^ T cells	41.7 ± 9.81	42.1 ± 9.19	41.2 ± 10.6	0.708
CD8^+^ T cells	23.6 (17.4, 29.4)	23.1 (17.5, 28.3)	24.1 (16.7, 31.0)	0.858
CD4^+^CD25^+^ T cells	19.9 (14.5, 25.8)	20.6 (16.1, 25.5)	19.3 (14.1, 25.8)	0.492
CD8^+^CD25^+^ T cells	2.01 (1.11, 3.41)	2.01 (1.00, 3.19)	2.00 (1.49, 3.79)	0.227
Activated CD4^+^ T cells	2.00 (1.16, 2.71)	1.80 (1.22, 2.56)	2.05 (1.11, 2.93)	0.519
Activated CD8^+^ T cells	3.03 (2.32, 4.78)	3.12 (2.00, 5.03)	2.99 (2.40, 4.20)	0.880
Naïve CD4^+^ T cells	17.2 (12.8, 23.6)	15.8 (13.2, 28.6)	18.3 (12.6, 20.2)	0.978
Naïve CD8^+^ T cells	21.1 (17.4, 29.4)	22.5 (18.1, 29.5)	19.4 (15.3, 28.5)	0.258
Memory CD4^+^ T cells	32.1 ± 8.60	31.5 ± 7.76	32.7 ± 9.53	0.558
Memory CD8^+^ T cells	15.1 (11.8, 21.9)	15.5 (12.5, 20.9)	14.8 (11.6, 23.0)	0.991
CD4^+^ CD28^+^ T cells	37.9 (32.2, 44.4)	39.5 (32.2, 45.5)	37.5 (32.2, 43.6)	0.816
CD8^+^ CD28^+^ T cells	13.6 (10.4, 17.6)	13.0 (9.39, 16.3)	14.5 (11.8, 19.4)	0.071
CD4^+^ CD95^+^ T cells	33.1 ± 9.19	33.1 ± 9.52	33.0 ± 8.94	0.965
CD8^+^ CD95^+^ T cells	25.1 (17.1, 31.5)	25.1 (15.8, 27.5)	24.0 (19.0, 32.4)	0.131

DKD: diabetic kidney disease, NLR: neutrophil-to-lymphocyte ratio, MLR: monocyte-to-lymphocyte ratio; PLR: platelet-to-lymphocyte ratio, NK cells: CD3^−^CD16^+^CD56^+^, activated CD4^+^ T cells: CD4^+^CD69^+^, activated CD8^+^ T cells: CD8^+^CD69^+^, Naïve CD4^+^ T cells: CD4^+^CD45RA^+^, Naïve CD8^+^ T cells: CD8^+^CD45RA^+^, memory CD4^+^ T cells: CD4^+^CD45RO^+^, memory CD8^+^ T cells: CD8^+^CD45RO^+^. * *p* < 0.05.

**Table 3:** Univariate and multivariate Cox analyses of factors associated with overall survival in patients with DKD.

Variables	Univariate Analysis		Lasso-Multivariate Analysis	
	HR (95% CI)	*p* Value	HR (95% CI)	*p* Value
Age	0.990 (0.904, 1.084)	0.821		
Men	0.281 (0.037, 2.153)	0.222		
Hypertension	Not estimable ^#^	0.998		
Duration of diabetes	1.022 (0.957, 1.092)	0.512		
Hemoglobin	0.978 (0.953, 1.004)	0.100		
Lymphocytes	0.790 (0.258, 2.413)	0.679		
NLR	1.312 (0.973, 1.769)	0.075 *		
MLR	1.903 (0.089, 40.630)	0.680		
PLR	1.010 (1.003, 1.016)	0.004 *	1.009 (1.002, 1.016)	0.013 *
Immunoglobin M	1.073 (0.460, 2.507)	0.870		
HbA1c	0.761 (0.530, 1.091)	0.138		
Urinary β2 microglobulin	0.990 (0.968, 1.013)	0.402		
SCr	1.012 (1.004, 1.021)	0.005 *	1.009 (1.000, 1.019)	0.047 *
BUN	1.089 (1.020, 1.162)	0.011 *		
Cystatin C	1.395 (0.717, 2.715)	0.327		
eGFR _CKD-EPI_	0.974 (0.947, 1.002)	0.068 *		
ACr	1.000 (1.000,1.000)	0.562		
CD3^+^ T cells	1.020 (0.959, 1.085)	0.533		
CD19^+^ B cells	0.967 (0.874, 1.070)	0.518		
NK cells	1.000 (0.945, 1.058)	0.998		
CD5^+^ B cells	0.775 (0.564, 1.065)	0.116		
CD5^−^ B cells	1.007 (0.876, 1.158)	0.918		
CD4^+^ T cells	1.036 (0.975, 1.100)	0.258		
CD8^+^ T cells	0.967 (0.904, 1.035)	0.334		
CD4^+^CD25^+^ T cells	0.929 (0.855, 1.008)	0.078 *	0.931 (0.848, 1.023)	0.136
CD8^+^CD25^+^ T cells	1.030 (0.654, 1.622)	0.898		
Activated CD4^+^ T cells	1.069 (0.699, 1.636)	0.758		
Activated CD8^+^ T cells	0.937 (0.757, 1.159)	0.546		
Naïve CD4^+^ T cells	0.971 (0.910, 1.035)	0.366		
Naïve CD8^+^ T cells	1.029 (0.965, 1.098)	0.384		
Memory CD4^+^ T cells	1.002 (0.934, 1.075)	0.954		
Memory CD8^+^ T cells	1.045 (0.966, 1.130)	0.271		
CD4^+^ CD28^+^ T cells	0.970 (0.911, 1.033)	0.347		
CD8^+^ CD28^+^ T cells	1.046 (0.952, 1.149)	0.351		
CD4^+^ CD95^+^ T cells	0.980 (0.924, 1.040)	0.511		
CD8^+^ CD95^+^ T cells	1.002 (0.939, 1.071)	0.943		

NLR: neutrophil-to-lymphocyte ratio, MLR: monocyte-to-lymphocyte ratio, PLR: platelet-to-lymphocyte ratio, SCr: serum creatinine, BUN: blood urea nitrogen, ACr; albumin-to-creatinine ratio, eGFR: estimated glomerular filtration rate, NK cells: CD3^−^CD16^+^CD56^+^, activated CD4^+^ T cells: CD4^+^CD69^+^, activated CD8^+^ T cells: CD8^+^CD69^+^, Naïve CD4^+^ T cells: CD4^+^CD45RA^+^, Naïve CD8^+^ T cells: CD8^+^CD45RA^+^, memory CD4^+^ T cells: CD4^+^CD45RO^+^, memory CD8^+^ T cells: CD8^+^CD45RO^+^. * *p* < 0.1. ^#^ The HR for hypertension was not estimable because of quasi-complete separation.

**Table 4:** Collinearity analysis of six candidate prognostic variables in patients with DKD.

Variables	Before Lasso Analysis		After Lasso Analysis	
	VIF	Tolerance	VIF	Tolerance
NLR	2.321	0.431		
PLR	1.984	0.504	1.143	0.875
SCr	7.384	0.135	1.164	0.859
BUN	1.835	0.545		
eGFR_CKD-EPI_	7.337	0.136		
CD4^+^CD25^+^ T cells	1.099	0.910	1.041	0.961

SCr: serum creatinine, BUN: blood urea nitrogen, eGFR: estimated glomerular filtration rate, NLR: neutrophil-to-lymphocyte ratio, PLR: platelet-to-lymphocyte ratio, VlF: variance infation factor.

**Table 5:** Cox analysis of factors associated with overall survival in the whole T2DM cohort.

Variables	Lasso-Multivariate Analysis	
	HR (95% CI)	*p* Value
PLR	1.008 (1.001, 1.014)	0.017 *
SCr	1.013 (1.006, 1.020)	<0.001 *
CD4^+^CD25^+^ T cells	0.920 (0.858, 0.986)	0.019 *

PLR: platelet-to-lymphocyte ratio, SCr: serum creatinine. * *p* < 0.05.

**Table 6:** Patients’ characteristics of the two groups with distinct prognostic features (*p* < 0.1).

Variables	Low-Risk Group	High-Risk Group	*p* Value
Cases	50	24	
DKD	17 (34.0%)	22 (91.7%)	<0.001 *
Hb (g/L)	126 ± 17.1	113 ± 23.5	0.016 *
Lymphocytes (10^9^/L)	1.71 (1.37, 2.12)	1.24 (0.92, 1.54)	<0.001 *
Platelets (10^9^/L)	176 (150, 213)	213 (173, 262)	0.032 *
Alb (g/L)	35.7 ± 3.02	33.9 ± 3.59	0.047 *
Urinary β2 microglobulin (mg/L)	0.48 (0.12, 2.66)	9.59 (1.05, 33.9)	<0.001 *
SCr (μmol/L)	77.9 (69.2, 88.5)	168 (136, 212)	<0.001 *
BUN (mmol/L)	5.47 (4.43, 7.62)	11.5 (9.64, 15.3)	<0.001 *
Cystatin C (mg/L)	1.17 (0.99, 1.51)	2.55 (2.04, 3.03)	<0.001 *
eGFR _CKD-EPI_ (ml/min/1.73 m^2^)	75.8 (67.4, 85.6)	31.1 (19.7, 40.1)	<0.001 *
ACr (mg/g Cr)	23.9 (6.29, 47.4)	500 (91.7, 1512)	<0.001 *
NLR	2.20 (1.69, 2.78)	3.68 (2.81, 5.20)	<0.001 *
MLR	0.30 (0.23, 0.38)	0.48 (0.36, 0.68)	<0.001 *
PLR	106 (87.6, 134)	174 (128, 256)	<0.001 *
NK cells	15.1 (11.2, 19.1)	18.8 (16.2, 25.8)	0.011 *
CD5^+^ B cells	1.65 (0.87, 4.49)	1.11 (0.40, 2.30)	0.036 *
CD4^+^CD25^+^ T cells	21.1 (15.2, 27.5)	17.4 (13.4, 23.1)	0.078
Naïve CD8^+^ T cells	19.5 (16.1, 23.7)	25.0 (20.7, 33.3)	0.007 *

DKD: diabetic kidney disease; Hb, hemoglobin; Alb, serum albumin; SCr, serum creatinine; BUN, blood urea nitrogen; eGFR, estimated glomerular filtration rate; ACr, albumin-to-creatinine ratio; * *p* < 0.05.

**Table 7:** Summary of exposures for Mendelian randomization.

Analytical Objective	OpenGWAS ID	Protein Expression Trait	Sample Size
Baseline Assessment: Assesses the generic effect of CD4 or CD25 expression level.	ebi-a-GCST90002022	CD4 on CD4^+^ T cell	2912
	ebi-a-GCST90001960	CD25 on CD4^+^ T cell	2920
Identify Causal Cell Type: Treg; Naïve conventional T cell; effector conventional T cell	ebi-a-GCST90001936	CD25 on CD4 regulatory T cell	3435
	ebi-a-GCST90001934	CD25 on CD45RA^+^ CD4 not regulatory T cell	3435
	ebi-a-GCST90001933	CD25 on CD45RA^−^ CD4 not regulatory T cell	3435
Treg Functional State	ebi-a-GCST90001937	CD25 on resting CD4 regulatory T cell	3434
	ebi-a-GCST90001941	CD25 on secreting CD4 regulatory T cell	3435
	ebi-a-GCST90001939	CD25 on activated CD4 regulatory T cell	3435
	ebi-a-GCST90002066	CD4 on activated CD4 regulatory T cell	2920
Control for general T cell activation status	ebi-a-GCST90001959	CD4 on HLA DR^+^ CD4^+^ T cell	3060
	ebi-a-GCST90002114	HLA DR on HLA DR^+^ CD4^+^ T cell	3060

## Data Availability

The datasets generated and analyzed during the current study are available from the corresponding author on request.

## Supplementary Materials

The following supporting information can be downloaded at https://www.mdpi.com/article/10.3390/biomedicines14051171/s1, Table S1: Univariate and multivariate Cox analyses of factors associated with overall survival in the whole T2DM cohort. Table S2: Patients’ characteristics of the two groups with distinct prognostic features. Table S3: Collinearity analysis of the 9 indicators included after univariate analysis in patients with T2DM. Table S4: Number of SNPs and instrument strength for immune phenotypes used in MR analysis. Figure S1: Feature selection using LASSO regression for prognostic model development in patients with T2DM. Figure S2: Validation of the prognostic nomogram for survival prediction in patients with T2DM.
