# Supplementary material for: Circulating Lymphocyte Subsets Are Associated with Diabetic Kidney Disease and Overall Survival in Patients with Type 2 Diabetes
Source: Biomedicines. 2026 May 21;14(5):1171. doi: 10.3390/biomedicines14051171 (PMC13204377; doi:10.3390/biomedicines14051171)
Supplement: Supplementary file 1 [file biomedicines-14-01171-s001.zip › Supplementary Figure 1 legend.pdf]

Supplementary Figure S1. Feature selection using LASSO regression for prognostic model development in patients with T2DM. (A) 5 risk factors selected using LASSO regression analysis. The optimal tuning parameter (λ) was determined via ten-fold cross-validation based on the minimum criteria. The vertical dashed lines indicate the optimal λ values selected by the minimum criteria and the one-standard-error (1-SE) rule, which provides the most parsimonious model within one standard error of the minimum. (B) LASSO coefficient profiles of the 9 features. A coefficient profile plot was produced against the log (λ) sequence. At minimum criteria including PLR, HbA1c, SCr, BUN and $CD4^{+}CD25^{+}$ T cells.
