# Supplementary material for: Circulating Lymphocyte Subsets Are Associated with Diabetic Kidney Disease and Overall Survival in Patients with Type 2 Diabetes
Source: Biomedicines. 2026 May 21;14(5):1171. doi: 10.3390/biomedicines14051171 (PMC13204377; doi:10.3390/biomedicines14051171)
Supplement: Supplementary file 1 [file biomedicines-14-01171-s001.zip › Supplementary Figure 2 legend.pdf]

Supplementary Figure S2. Validation of the prognostic nomogram for survival prediction in patients with T2DM.
(A-C) Calibration curves for 2-year (A), 3-year (B) and 5-year (C) overall survival predictions. The x-axis shows nomogram-predicted probabilities, while the y-axis represents observed outcomes. Diagonal dashed lines indicate ideal calibration, where predictions perfectly match actual survival.
(D) Time-dependent receiver operating characteristic (ROC) curves for 1-, 2-, 3- and 5-year survival predictions.
(E) Temporal evolution of area under the curve (AUC) values across the follow-up period.
(F) Bootstrap internal validation (1,000 resamples) demonstrating model robustness. Gray lines represent individual bootstrap ROC curves; the blue line shows the mean ROC curve.
(G-I) Decision curve analysis evaluating clinical utility of the nomogram. Net benefit analysis for (A) 2-year; (B) 3-year; and (C) 5-year survival predictions. The y-axis represents net benefit, while the x-axis shows threshold probabilities. Blue lines depict nomogram performance; red lines indicate treat-all (upper) and green lines indicate treat-none (lower) strategies. The region between vertical dashed lines indicates threshold probabilities where the nomogram provides superior net benefit compared to both default strategies. The model provided superior net benefit compared to treat-all or treat-none strategies when decision thresholds exceeded 4%. Clinical utility spans of this anaysis were defined as: 2-year predictions (thresholds: 0.04-0.38), 3-year predictions (0.04-0.69), and 5-year predictions (>0.04).
