# Supplementary material for: Circulating Lymphocyte Subsets Are Associated with Diabetic Kidney Disease and Overall Survival in Patients with Type 2 Diabetes
Source: Biomedicines. 2026 May 21;14(5):1171. doi: 10.3390/biomedicines14051171 (PMC13204377; doi:10.3390/biomedicines14051171)
Supplement: Supplementary file 1 [file biomedicines-14-01171-s001.zip › Supplementary Table 1.pdf]

Supplementary Table S1. Univariate and multivariate Cox analyses of factors associated with overall survival in the whole T2DM cohort

Variables	Univariate analysis		Lasso-Multivariate analysis	
	**HR (95% CI)**	***P* Value**	**HR (95% CI)**	***P* Value**
Age	1.062 (0.991,1.139)	0.090		
Men	0.778 (0.226,2.673)	0.690		
Hypertension	2.655 (0.844,8.355)	0.095		
Duration of diabetes	1.044 (0.997,1.094)	0.070		
**Hemoglobin**	**0.967 (0.948,0.986)**	**<0.001***		
Lymphocytes	1.035 (0.538,1.993)	0.917		
**NLR**	**1.386 (1.006, 1.803)**	**0.015***		
MLR	3.382 (0.237, 48.320)	0.369		
**PLR**	**1.011 (1.005, 1.017)**	**<0.001***	**1.008 (1.001, 1.014)**	**0.017***
Immunoglobin M	1.014 (0.446,2.304)	0.973		
**HbA1c**	**0.763 (0.662,0.878)**	**<0.001***		
Urinary β2 microglobulin	1.001 (0.983,1.019)	0.900		
**SCr**	**1.015 (1.008,1.021)**	**<0.001***	**1.013 (1.006, 1.020)**	**<0.001***
**BUN**	**1.127 (1.069,1.190)**	**<0.001***		
**Cystatin C**	**1.795 (1.167,2.760)**	**0.008***		
**eGFR $_{CKD-EPI}$**	**0.968 (0.951,0.985)**	**<0.001***		
ACr	1.000 (0.999,1.000)	0.686		
$CD3^+$ T cells	1.004 (0.964,1.045)	0.856		
$CD19^+$ B cells	0.998 (0.947,1.053)	0.953		
NK cells	1.021 (0.971,1.073)	0.415		
$CD5^+$ B cells	1.011 (0.957,1.067)	0.700		
$CD5^-$ B cells	0.949 (0.837,1.077)	0.418		
$CD4^+$ T cells	1.015 (0.969,1.063)	0.535		
$CD8^+$ T cells	0.984 (0.937,1.033)	0.519		
**$CD4^+CD25^+$ T cells**	**0.930 (0.872,0.992)**	**0.028***	**0.920 (0.858, 0.986)**	**0.019***
$CD8^+CD25^+$ T cells	0.924 (0.744,1.147)	0.473		
Activated $CD4^+$ T cells	0.924 (0.730,1.171)	0.515		
Activated $CD8^+$ T cells	1.017 (0.850,1.216)	0.857		
Naïve $CD4^+$ T cells	0.988 (0.944,1.035)	0.611		
Naïve $CD8^+$ T cells	1.038 (0.993,1.084)	0.097		
Memory $CD4^+$ T cells	0.998 (0.953,1.045)	0.942		
Memory $CD8^+$ T cells	1.040 (0.987,1.097)	0.143		
$CD4^+$ $CD28^+$ T cells	0.981 (0.940,1.023)	0.364		
$CD8^+$ $CD28^+$ T cells	1.028 (0.954,1.107)	0.467		
$CD4^+$ $CD95^+$ T cells	0.968 (0.917,1.021)	0.234		
$CD8^+$ $CD95^+$ T cells	1.004 (0.957,1.053)	0.878		

NLR: Neutrophil-to-lymphocyte ratio, MLR: Monocyte-to-lymphocyte ratio, PLR: Platelet-to-lymphocyte ratio, SCr: serum creatinine, BUN: blood urea nitrogen, ACr; albumin-to-creatinine ratio, eGFR: estimated glomerular filtration rate, NK cells: $CD3^-CD16^+CD56^+$, Activated $CD4^+$ T cells: $CD4^+CD69^+$, Activated $CD8^+$ T cells: $CD8^+CD69^+$, Naïve $CD4^+$ T cells: $CD4^+CD45RA^+$, Naïve $CD8^+$ T cells: $CD8^+CD45RA^+$, Memory $CD4^+$ T cells: $CD4^+CD45RO^+$, Memory $CD8^+$ T cells: $CD8^+CD45RO^+$. *P<0.05.
