# Supplementary material for: Circulating Lymphocyte Subsets Are Associated with Diabetic Kidney Disease and Overall Survival in Patients with Type 2 Diabetes
Source: Biomedicines. 2026 May 21;14(5):1171. doi: 10.3390/biomedicines14051171 (PMC13204377; doi:10.3390/biomedicines14051171)
Supplement: Supplementary file 1 [file biomedicines-14-01171-s001.zip › Supplementary Table 2.pdf]

**Supplementary Table S2.** Patients' characteristics of the two groups with distinct prognostic features.

| Variables | Low-risk group | High-risk group | P Value |
|---|---|---|---|
| Cases | 50 | 24 | |
| DKD | 17 (34.0%) | 22 (91.7%) | <0.001* |
| Age (years) | 82.0 (77.0, 87.0) | 84.0 (81.0, 88.0) | 0.320 |
| Men, n (%) | 39 (78.0%) | 21 (87.5%) | 0.527 |
| Hypertension, n (%) | 45 (90.0%) | 23 (95.8%) | 0.518 |
| Duration of diabetes (years) | 10.0 (4.25, 20.0) | 14.0 (9.50, 20.0) | 0.305 |
| Hb (g/L) | 126 ±17.1 | 113 ±23.5 | 0.016* |
| White blood cells ($10^9$/L) | 6.11 (5.42, 7.10) | 5.89 (5.62, 7.20) | 0.725 |
| Neutrophil ($10^9$/L) | 3.64 (3.03, 4.49) | 4.12 (3.56, 5.27) | 0.062 |
| Lymphocytes ($10^9$/L) | 1.71 (1.37, 2.12) | 1.24 (0.92, 1.54) | <0.001* |
| Monocytes ($10^9$/L) | 0.49 (0.41, 0.60) | 0.56 (0.46, 0.73) | 0.089 |
| Platelets ($10^9$/L) | 176 (150, 213) | 213 (173, 262) | 0.032* |
| IgA (g/L) | 2.75 (1.61, 3.99) | 2.25 (1.80, 2.99) | 0.323 |
| IgG (g/L) | 12.2 (10.4, 14.9) | 11.3 (10.0, 12.5) | 0.177 |
| IgM (g/L) | 0.59 (0.37, 0.88) | 0.58 (0.46, 0.89) | 0.712 |
| HbA1c (%) | 6.90 (6.10, 7.97) | 7.00 (6.27, 7.53) | 0.982 |
| Alb (g/L) | 35.7 ±3.02 | 33.9 ±3.59 | 0.047* |
| Urinary β2 microglobulin (mg/L) | 0.48 (0.12, 2.66) | 9.59 (1.05, 33.9) | <0.001* |
| Uric acid (μmol/L) | 361 ±88.6 | 400 ±107 | 0.131 |
| SCr (μmol/L) | 77.9 (69.2, 88.5) | 168 (136, 212) | <0.001* |
| BUN (mmol/L) | 5.47 (4.43, 7.62) | 11.5 (9.64, 15.3) | <0.001* |
| Cystatin C (mg/L) | 1.17 (0.99, 1.51) | 2.55 (2.04, 3.03) | <0.001* |
| eGFR $_{CKD-EPI}$ (ml/min/1.73m2) | 75.8 (67.4, 85.6) | 31.1 (19.7, 40.1) | <0.001* |
| ACr (mg/g Cr) | 23.9 (6.29, 47.4) | 500 (91.7, 1512) | <0.001* |
| NLR | 2.20 (1.69, 2.78) | 3.68 (2.81, 5.20) | <0.001* |
| MLR | 0.30 (0.23, 0.38) | 0.48 (0.36, 0.68) | <0.001* |
| PLR | 106 (87.6, 134) | 174 (128, 256) | <0.001* |
| $CD3^+$ T cells | 73.0 (64.9, 77.3) | 69.5 (67.8, 74.0) | 0.307 |
| $CD19^+$ B cells | 7.44 (4.54, 12.4) | 6.50 (2.83, 10.4) | 0.123 |
| NK cells | 15.1 (11.2, 19.1) | 18.8 (16.2, 25.8) | 0.011* |
| $CD5^+$ B cells | 1.65 (0.87, 4.49) | 1.11 (0.40, 2.30) | 0.036* |
| $CD5^-$ B cells | 5.02 (3.28, 7.96) | 4.18 (2.05, 8.74) | 0.362 |
| $CD4^+$ T cells | 41.1 ±9.59 | 43.0 ±10.3 | 0.446 |
| $CD8^+$ T cells | 24.5 (17.6, 31.1) | 21.3 (17.3, 27.9) | 0.453 |
| $CD4^+CD25^+$ T cells | 21.1 (15.2, 27.5) | 17.4 (13.4, 23.1) | 0.078 |
| $CD8^+CD25^+$ T cells | 2.09 (1.25, 3.56) | 1.83 (1.04, 3.14) | 0.353 |
| Activated $CD4^+$ T cells | 2.00 (1.12, 2.83) | 1.99 (1.34, 2.45) | 0.936 |
| Activated $CD8^+$ T cells | 2.80 (2.22, 4.02) | 4.20 (2.92, 5.10) | 0.066 |
| Naïve $CD4^+$ T cells | 17.6 (14.1, 21.4) | 15.5 (9.77, 28.6) | 0.338 |
| Naïve $CD8^+$ T cells | 19.5 (16.1, 23.7) | 25.0 (20.7, 33.3) | 0.007* |
| Memory $CD4^+$ T cells | 33.1 ±8.54 | 30.0 ±8.53 | 0.149 |
| Memory $CD8^+$ T cells | 14.8 (11.7, 21.9) | 17.0 (13.1, 21.7) | 0.607 |
| $CD4^+$ $CD28^+$ T cells | 39.5 (33.3, 45.7) | 36.1 (30.2, 40.1) | 0.125 |
| $CD8^+$ $CD28^+$ T cells | 13.2 (10.9, 17.5) | 14.2 (9.46, 17.5) | 0.760 |
| $CD4^+$ $CD95^+$ T cells | 33.8 ±9.33 | 31.7 ±8.92 | 0.365 |
| $CD8^+$ $CD95^+$ T cells | 22.8 (17.1, 31.7) | 26.7 (20.4, 30.8) | 0.429 |

DKD: diabetic kidney disease; Hb, hemoglobin; IgA, immunoglobulin A; IgG, immunoglobulin G; IgM, immunoglobulin M; HbA1c, glycosylated hemoglobin; Alb, serum albumin; SCr, serum creatinine; BUN,

blood urea nitrogen; ACr, albumin-to-creatinine ratio; eGFR, estimated glomerular filtration rate; *P<0.05.
