# Supplementary material for: Circulating Lymphocyte Subsets Are Associated with Diabetic Kidney Disease and Overall Survival in Patients with Type 2 Diabetes
Source: Biomedicines. 2026 May 21;14(5):1171. doi: 10.3390/biomedicines14051171 (PMC13204377; doi:10.3390/biomedicines14051171)
Supplement: Supplementary file 1 [file biomedicines-14-01171-s001.zip › Supplementary Table 3.pdf]

Supplementary Table S3. Collinearity analysis of the 9 indicators included after univariate analysis in patients with T2DM

Variables	Before Lasso analysis		After Lasso analysis	
	**VIF**	**Tolerance**	**VIF**	**Tolerance**
Hemoglobin	1.114	0.898		
NLR	2.604	0.384		
PLR	2.361	0.423	1.235	0.810
HbA1c	1.772	0.564	1.029	0.971
SCr	6.945	0.144	2.459	0.407
BUN	2.314	0.432	2.267	0.441
Cystatin C	4.742	0.211		
eGFR $_{CKD\text{-}EPI}$	9.108	0.110		
$CD4^{+}CD25^{+}$ T cells	1.027	0.974	1.011	0.989

SCr: serum creatinine, BUN: blood urea nitrogen, eGFR: estimated glomerular filtration rate, NLR: Neutrophil-to-lymphocyte ratio, PLR: Platelet-to-lymphocyte ratio, VIF: variance infation factor.
