# Supplementary material for: Circulating Lymphocyte Subsets Are Associated with Diabetic Kidney Disease and Overall Survival in Patients with Type 2 Diabetes
Source: Biomedicines. 2026 May 21;14(5):1171. doi: 10.3390/biomedicines14051171 (PMC13204377; doi:10.3390/biomedicines14051171)
Supplement: Supplementary file 1 [file biomedicines-14-01171-s001.zip › Supplementary Table 4.pdf]

Supplementary Table S4. Number of SNPs and instrument strength for immune phenotypes used in MR analysis.

Immune phenotype	GWAS ID	SNPs after clumping	SNPs used in MR	Mean F statistic	Minimum F statistic
CD4 on activated CD4 regulatory T cells	ebi-a-GCST90002066	3	3	45.66	34.87
CD4 on CD4+ T cells	ebi-a-GCST90002022	4	3	53.54	36.80
CD4 on HLA-DR+ CD4+ T cells	ebi-a-GCST90001959	2	2	38.22	35.71
CD25 on CD45RA- CD4 non-regulatory T cells	ebi-a-GCST90001933	2	2	77.70	33.38
CD25 on CD45RA+ CD4 non-regulatory T cells	ebi-a-GCST90001934	1	1	58.51	58.51
CD25 on resting CD4 regulatory T cells	ebi-a-GCST90001937	1	1	41.90	41.90
CD25 on secreting CD4 regulatory T cells	ebi-a-GCST90001941	2	1	42.46	42.46
CD25 on CD4+ T cells	ebi-a-GCST90001960	1	1	46.02	46.02
HLA-DR on HLA-DR+ CD4+ T cells	ebi-a-GCST90002114	1	1	36.39	36.39
