# Supplementary figures and images for: Circulating Lymphocyte Subsets Are Associated with Diabetic Kidney Disease and Overall Survival in Patients with Type 2 Diabetes
Source: Biomedicines. 2026 May 21;14(5):1171. doi: 10.3390/biomedicines14051171 (PMC13204377; doi:10.3390/biomedicines14051171)

**A**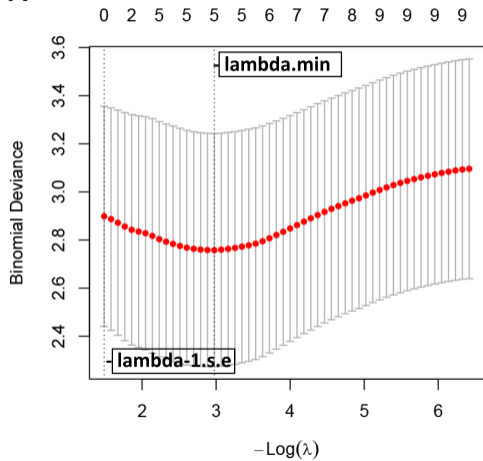**B**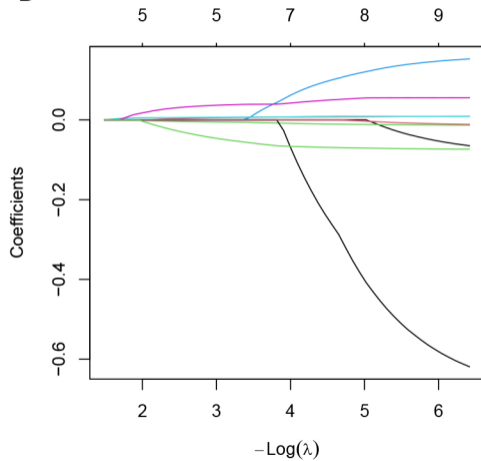

Supplement: Supplementary file 1 [file biomedicines-14-01171-s001.zip › Supplementary Figure 1.pdf]

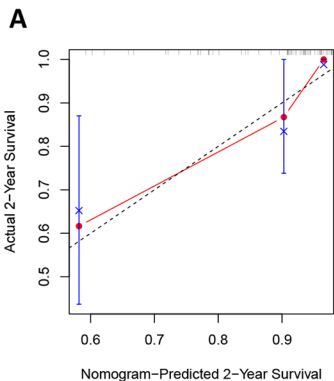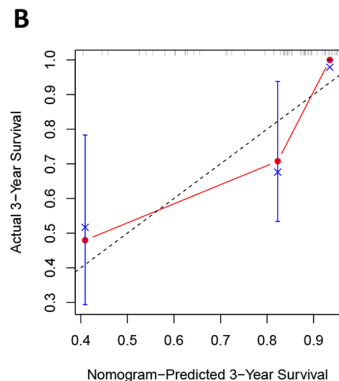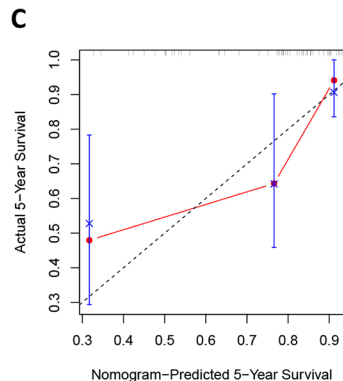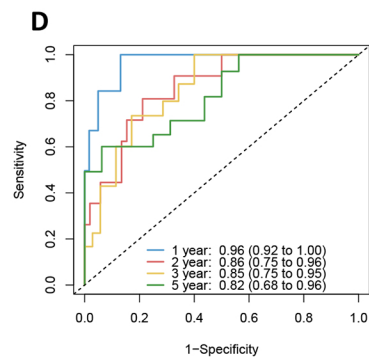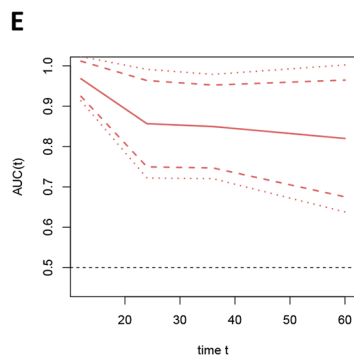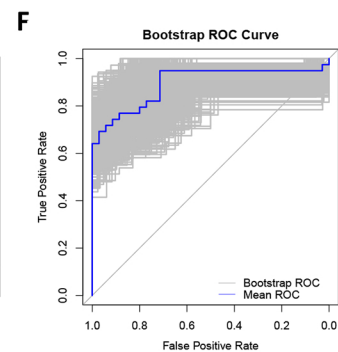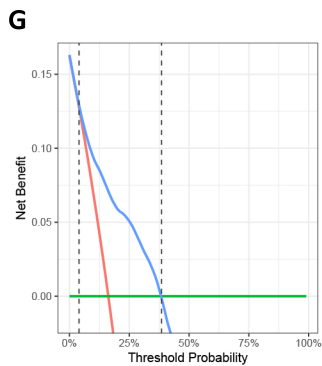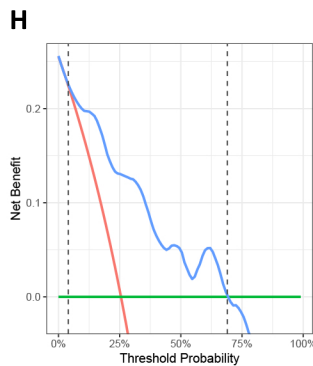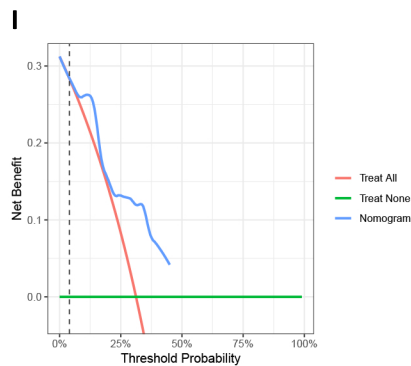

Supplement: Supplementary file 1 [file biomedicines-14-01171-s001.zip › Supplementary Figure 2.pdf]
